# The Ticking Surgical Clock: Outcome, Predictor, or a Bit of Both?

**DOI:** 10.21470/1678-9741-2024-0084

**Published:** 2025-02-05

**Authors:** Rohan Magoon, Jaffrey Kalaiselvan, Jes Jose

**Affiliations:** 1 Department of Cardiac Anaesthesia, Atal Bihari Vajpayee Institute of Medical Sciences (ABVIMS) and Dr. Ram Manohar Lohia Hospital, New Delhi, India; 2 Department of Cardiac Anaesthesia, Atal Bihari Vajpayee Institute of Medical Sciences (ABVIMS) and Dr. Ram Manohar Lohia Hospital, New Delhi, India; 3 Department of Cardiac Anaesthesiology, Sri Jayadeva Institute of Cardiovascular Sciences and Research, Bengaluru, Karnataka, India. E-mail: drjesjoseworkmail@gmail.com

To The Editor,

Jucá et al.^[1]^ recently subanalyzed the Registro Paulista de Cirurgia Cardiovascular (or REPLICCAR) II database in an endeavor to evaluate the prognostic potential of the time difference between the cardiopulmonary bypass (CPB) and the aortic cross-clamping durations (TDC-C). The research approach of the group pretty much regards the TDC-C as a ‘predictor of outcome’ in a study involving 3,090 patients undergoing coronary artery bypass grafting (CABG)^[1]^. Having said that, a discussion in the matter would be opportune to assist the readership interpret whether the former dynamic parameter features as an ‘outcome’, ‘predictor’, or a bit of both.

To begin with, the authors do acknowledge as to how a prolonged TDC-C can be potentially linked to the challenges in weaning patients from CPB^[1]^. In this context, it would have been worthwhile to know of the corresponding incidence of low cardiac output syndrome, diastolic dysfunction, or for that matter postoperative new-onset atrial fibrillation in their setting, which would have likely deemed the readiness to weaning from CPB difficult^[2,3,4]^. Of note here, the details on requirement of intra-aortic balloon pump (IABP) support would have been equally important considering a research frame involving an operative subset of CABG^[1,2]^. Talking of hemodynamic support, it is further thought-provoking to witness Monaco et al.^[2]^ integrate the vasoactive-inotropic score (VIS) in their decision-making algorithm pertaining to complex CPB separation. In a comprehensive review on the topic, they outline the categories of the ease of weaning stratified in accordance with VIS (< 10 being easy, with 10-30 being difficult, and > 30 being complex, often requiring IABP support)^[2]^. Besides, the prognostic implications of an elevated VIS can by itself not be undermined^[5]^. Finally, it is believed that alongside quantitative aspects relating to extracorporeal circulation, the qualitative attributes like the degree of hypothermia, perfusion dynamics, and the blood/blood product management could have had a simultaneous role to play in determining the contextual propensity to poor outcomes after CABG^[6]^. All said and done, the index study by Jucá et al.^[1]^ undoubtedly serves as a particularly useful template for future research in this subject of wide clinical interest to the cardiac surgical fraternity.

## References

[1] Jucá FG, Freitas FL, Goncharov M, Pes DL, Jucá MEC, Dalian LRP (2024). Difference between cardiopulmonary bypass time and aortic
cross-clamping time as a predictor of complications after coronary artery
bypass grafting. Braz J Cardiovasc Surg.

[2] Monaco F, Di Prima AL, Kim JH, Plamondon MJ, Yavorovskiy A, Likhvantsev V (2020). Management of challenging cardiopulmonary bypass
separation. J Cardiothorac Vasc Anesth.

[3] Bernard F, Denault A, Babin D, Goyer C, Couture P, Couturier A (2001). Diastolic dysfunction is predictive of difficult weaning from
cardiopulmonary bypass. Anesth Analg.

[4] Ashes CM, Yu M, Meineri M, Katznelson R, Carroll J, Rao V (2014). Diastolic dysfunction, cardiopulmonary bypass, and atrial
fibrillation after coronary artery bypass graft surgery. Br J Anaesth.

[5] Magoon R, Jose J (2022). Prognostic implications of quantifying haemodynamic support:
looking beyond a snapshot score. Braz J Cardiovasc Surg.

[6] Jegger D, Revelly JP, Horisberger J, von Segesser LK, Ruchat P (2007). Establishing an association between a peri-operative perfusion
score system (PerfSCORE) and post-operative patient morbidity/mortality
during CPB cardiac surgery. Perfusion.

